# Cytophagic Histiocytic Panniculitis and Kikuchi-Fujimoto-Like Lymphadenitis in a Patient With Systemic Lupus Erythematosus

**DOI:** 10.7759/cureus.28951

**Published:** 2022-09-08

**Authors:** Aref Moshayedi, Sofia Shea, Abhishek Nandan, Sarah Ford

**Affiliations:** 1 Dermatology, Virginia Commonwealth University School of Medicine, Richmond, USA; 2 Dermatopathology, Hunter Holmes McGuire Veterans Affairs Medical Center, Richmond, USA; 3 Dermatopathology, Virginia Commonwealth University School of Medicine, Richmond, USA; 4 Rheumatology, Hunter Holmes McGuire Veterans Affairs Medical Center, Richmond, USA; 5 Rheumatology, Virginia Commonwealth University School of Medicine, Richmond, USA

**Keywords:** histiocyte, lupus erythematosus panniculitis, lymphadenitis, kikuchi fujimoto’s disease, kikuchi, kikuchi-fujimoto with systemis lupus erythematosous, chp, cytophagic histiocytic panniculitis, systemic lupus erythromatosus, sle

## Abstract

A 54-year-old African American male with systemic lupus erythematosus and chronic alcoholic hepatitis presented with recurrent fever, pancytopenia, transaminitis, weight loss, and widespread violaceous tender plaques. Skin biopsy revealed hemophagocytic histiocytes leading to a diagnosis of cytophagic histiocytic panniculitis in the setting of lupus panniculitis. During workup, an axillary lymph node biopsy mimicked Kikuchi-Fujimoto's disease. Treatment with tapering high-dose glucocorticoids, mycophenolate mofetil, and hydroxychloroquine induced remission of the disease. We believe the comorbid conditions of Kikuchi-Fujimoto-like pathology and cytophagic histiocytic panniculitis have not been documented in the literature to date in a patient with systemic lupus erythematosus.

## Introduction

Cytophagic histiocytic panniculitis (CHP) has previously been described in association with System Lupus Erythematosus (SLE) and is an autoinflammatory condition characterized by recurrent fevers, hepatic dysfunction, pancytopenia, and coagulopathy. It often follows a universally fatal disease course, with recurrent exacerbations and hospital admissions despite aggressive immunosuppression [1]. Here we present an unusual case of CHP that was diagnosed after extensive multi-disciplinary workup that was successfully treated with a tapering course of glucocorticoids, hydroxychloroquine (HCQ), and mycophenolate mofetil (MMF). We conclude with a discussion of the diagnostic challenges that may arise in patients with numerous comorbid conditions that can confound decisions about diagnosis and management.

## Case presentation

**Figure 1:**
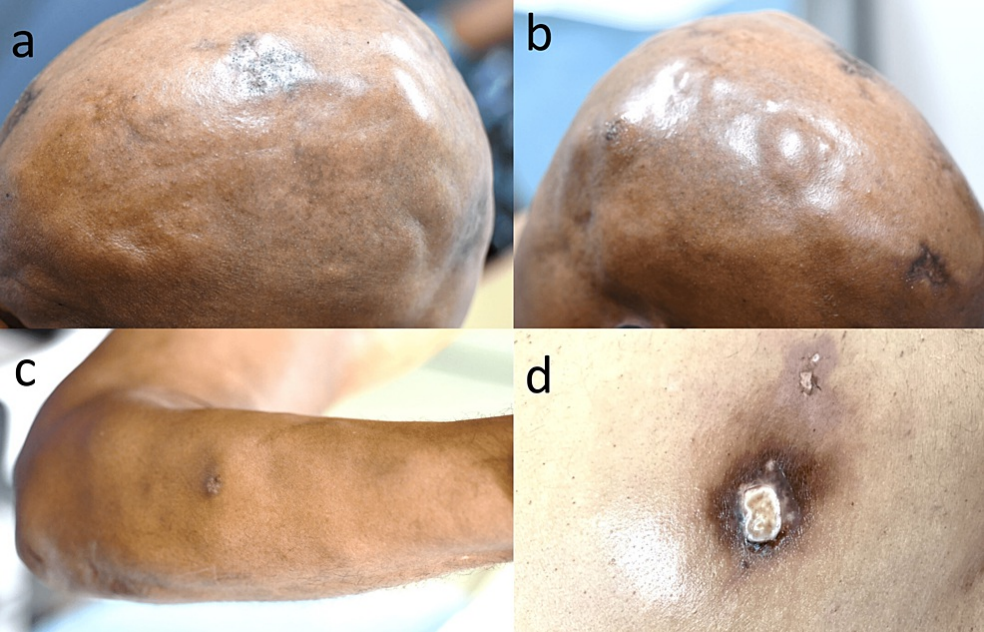
Photographs of the patient's tender, ulcerating nodules. A, B: Left and right parietal scalp (respectively) with hyperpigmented plaques and patches along with multiple scattered firm subcutaneous nodules. C: Multiple soft, rubbery, non-tender nodules on the right forearm with single ulceration at the dorsal mid-forearm. D: Firm, violaceous, tender plaque on the right posterior chest with multiple crusted erosions.

A 54-year-old African American male with a medical history significant for Systemic Lupus Erythematosus (SLE) and chronic alcoholic hepatitis presented to the rheumatology clinic with one month of recurrent fevers (38-39° C), pancytopenia, transaminitis, 30-pound weight loss, and tender, ulcerating nodules on the extremities, scalp, and back (Figures 1A-1D). His initial diagnosis of SLE was made 17 years before presentation at an outside facility, and at the time, he was found to have a positive antinuclear antibody (ANA) at 1:640. The patient’s SLE had been well controlled on 200 mg twice daily hydroxychloroquine for several years but had been discontinued one year before presentation due to unexplained transaminitis, intermittent leukopenia, and thrombocytopenia.

Initial workup showed pancytopenia (white blood cell count of 3.1x10^3/μL, hemoglobin of 13.1 g/dL, and 122,000 platelets/μL), transaminitis (aspartate aminotransferase of 278 IU/L, alanine aminotransferase of 289 IU/L), and normal erythrocyte sedimentation rate, C-reactive protein, Complement C3, and Complement C4. Serum haptoglobin was undetectable, and lactate dehydrogenase was elevated at 642 IU/L. A direct immunoglobin test was positive for a complement. Anti-double stranded DNA (dsDNA), anti-nuclear antibody (ANA), rheumatoid factor (RF), and antineutrophil cytoplasmic antibodies (ANCA) were all negative. An infectious workup was negative, including serologies for fungal and viral infections, Lyme disease, a hepatitis panel, blood cultures, urinalysis, and a chest radiograph.

Prednisone (40 mg) was initiated with a marked improvement in symptoms and complete cessation of fever. Over the next several weeks, however, high fever and worsening pancytopenia prompted an extended admission to the hospital at which point a chest computerized tomography (CT) scan revealed an enlarged left axillary lymph node. A positron emission tomography (PET) CT scan was notable for moderate metabolic activity in the left axillary lymph node and diffuse increased metabolic activity in subcutaneous tissue nodules. A left axillary lymph node biopsy revealed large numbers of CD163 positive histocytes and CD123 and CD25 positive plasmacytoid dendritic cells. The overall findings were consistent with a reactive lymph node and features of systemic lupus erythematosus lymphadenitis with Kikuchi-like features. A bone marrow biopsy was largely unrevealing and showed hypercellularity with maturing trilineage hematopoiesis and no increase in blasts.

**Figure 2:**
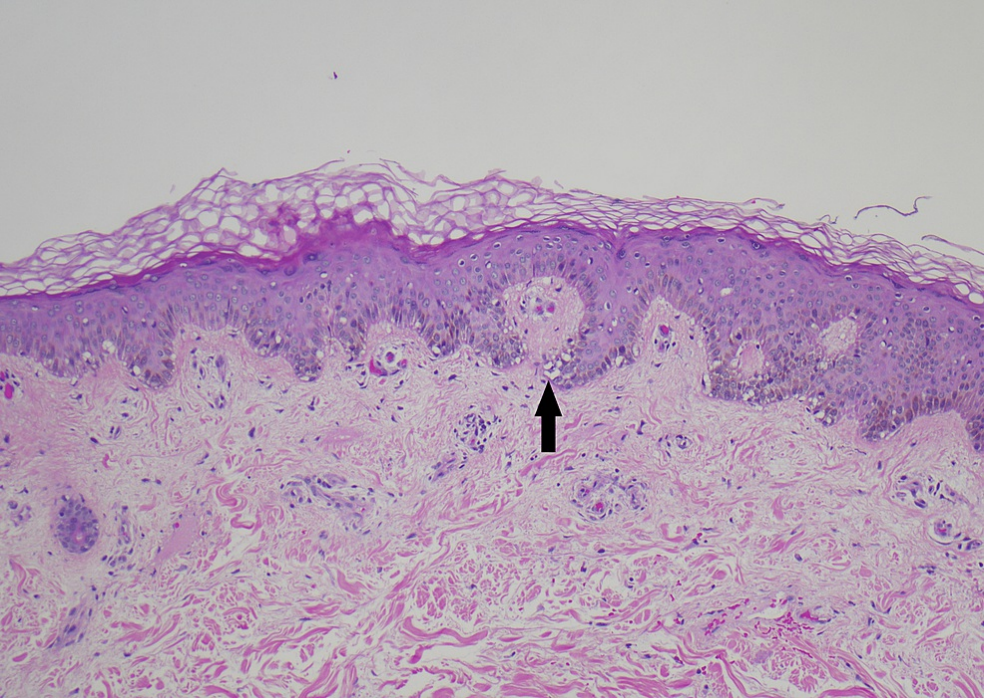
Low magnification view of epidermis demonstrating basovacular changes at the dermal-epidermal junction (arrow). (hematoxylin and eosin, 100x magnification).

**Figure 3:**
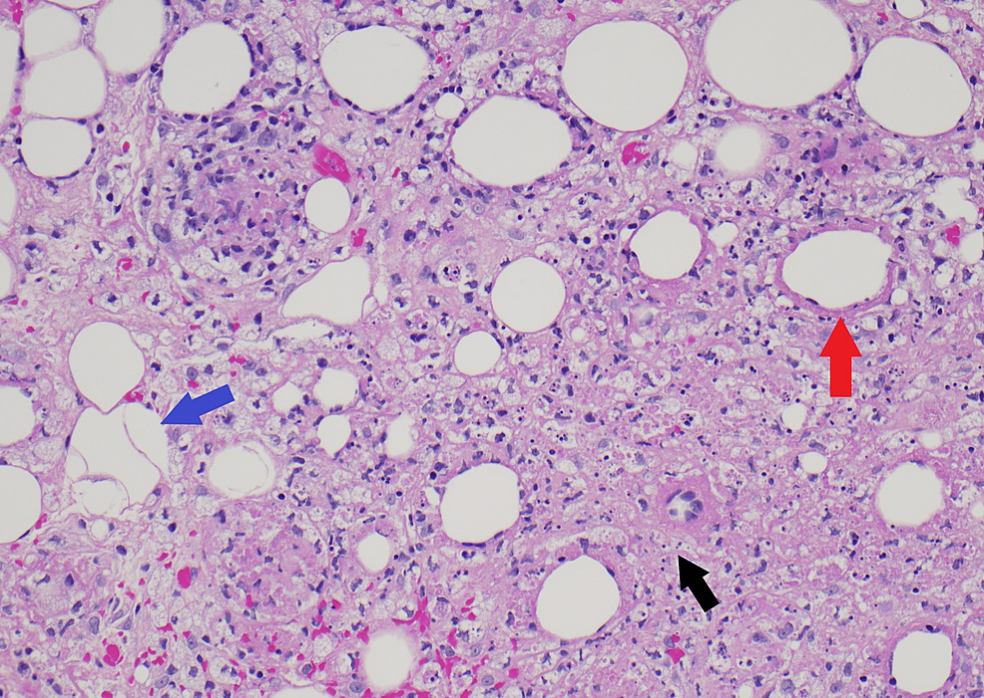
Several histologic features of lupus panniculitis are observed, including neutrophil-rich infiltrate (black arrow), hyalinization of fat globules (red arrow), and variable adipocyte size (blue arrow). (hematoxylin and eosin, 200x magnification).

**Figure 4:**
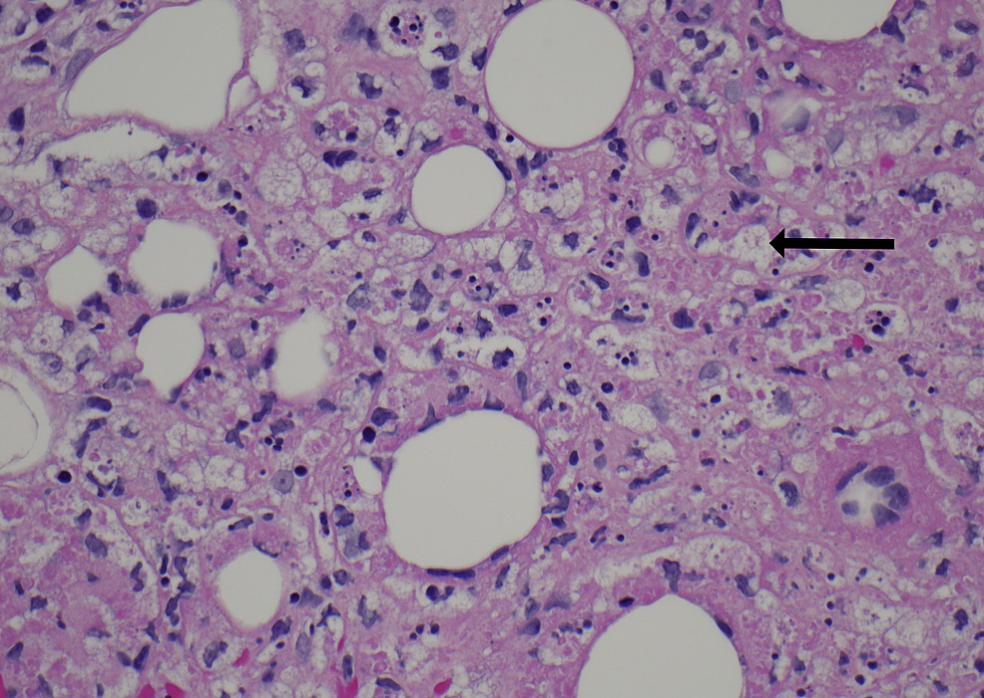
Diffuse fat necrosis of the subcutaneous tissue with clusters of histiocytes showing phagocytosis of red cells, white cells, and nuclear debris (arrow). (hematoxylin and eosin, 400x magnification)

Skin biopsies were obtained from the nodular lesions on the back, forearm, and scalp. Incisional biopsy of a nodule in the right posterior chest revealed hyaline necrosis of the subcutaneous tissue with neutrophil-rich infiltration and negative stains for microbes (Periodic acid-Schiff, Grocott's Methenamine Silver, acid-fast bacteria (AFB), and gram stains). A punch biopsy of a left forearm nodule was obtained, which revealed clusters of histiocytes showing phagocytosis of red cells, white cells, and nuclear debris. All three specimens demonstrated lobular panniculitis and extensive phagocytosis, consistent with a diagnosis of lupus panniculitis and CHP. Figure 2 demonstrates basovacular changes at the dermal-epidermal junction. Figures 3, 4 are higher magnification versions of the same biopsy revealing neutrophil-rich infiltrate and clusters of phagocytic histiocytes, respectively.

The patient was subsequently started on another prednisone taper starting at 40 mg, mycophenolate mofetil 1000 mg BID, and hydroxychloroquine 200 mg BID with subsequent improvement of fevers and skin lesions. He was maintained on hydroxychloroquine with no further flares or significant disease exacerbations.

## Discussion

CHP is a rare, distinct panniculitis disorder that is characterized histologically by infiltration of adipose tissue by benign cytophagic macrophages. CHP lies on the spectrum of autoinflammatory conditions that include hemophagocytic lymphohistiocytosis (HLH) [1]. CHP typically presents as ulcerating plaques and nodules that can persist or regress over weeks to months. Other clinical findings include fever, hepatosplenomegaly, and lymphadenopathy. Laboratory abnormalities can include pancytopenia, low/normal ESR, transaminitis, and coagulopathy. While rheumatologic disorders are known to be associated with hemophagocytic syndromes, CHP has rarely been reported in association with discoid or system lupus erythematosus [2-6].

In our patient, significant diagnostic uncertainty was present because of medical and social comorbidities. For instance, a previously established diagnosis of SLE without convincing evidence of active disease, Kikuchi-Fujimoto-like features on lymph node biopsy, and chronic alcoholic hepatitis all shared a significant overlap of disease features at presentation and confounded our diagnosis of CHP. Lupus panniculitis can also present similarly to CHP; therefore, differentiating the two disease processes via biopsy is essential because the former requires less intensive immunosuppression and is not associated with a potentially lethal disease course [7]. Additionally, our patient’s lymph node biopsy was notable for Kikuchi-Fujimoto-like pathology, which has been previously reported in association with SLE [8]. To our knowledge, this is the first case report of an individual with both Kikuchi-Fujimoto-like pathology and CHP.

The pathogenesis of CHP remains unclear, but it is assumed that cytokines secreted from dysregulated T cells activate macrophages and become cytophagic histiocytes [1]. Treatment of CHP is essential, given its potential for rapidly fatal clinical course and association with HLH [1]. After exclusion of infectious etiology, CHP must be managed with immunosuppressants, immunomodulators, or cytotoxic agents that will dampen cytokine release by activated lymphocytes, adipocytes, and macrophages that lead to the progressive destructive nature of the autoinflammatory disease. Previously reports have shown efficacy with the administration of high-dose glucocorticoids and cyclosporine A [2,3,9]. There has been a report of cyclosporine-resistant CHP that was successfully treated with tacrolimus [10]. An additional report has identified intravenous immunoglobulins (IVIG) and cyclophosphamide as efficacious [6]. In our patient, the excellent response was demonstrated with a combination of prednisone taper, MMF, and HCQ. Glucocorticoids and MMF were successfully withdrawn and the patient had no further relapses with an ongoing regimen of HCQ.

## Conclusions

We present a case of concurrent CHP, SLE, and Kikuchi-Fujimoto-like lymphadenitis in a 54-year-old male. To the best of our knowledge, the co-occurrence of these three clinical entities is rare and has not been documented in the literature to date. CHP has potentially fatal outcomes; therefore, clinical recognition and accurate diagnosis of CHP is paramount, especially considering that other clinical entities such as lupus panniculitis can present similarly. The mainstay of the management of CHP is the use of immunosuppressants, immunomodulators, or cytotoxic agents. Our patient went into remission with the administration of a prednisone taper, mycophenolate mofetil, and an ongoing regimen of hydroxychloroquine.
